# Great Debate: Should Surgery Be Omitted in the Management of Gastroesophageal Junction Cancers?

**DOI:** 10.1245/s10434-026-20293-0

**Published:** 2026-07-26

**Authors:** Meredith A. Gunder, Brian D. Badgwell, Kaitlyn J. Kelly

**Affiliations:** 1 Department of Surgery, Inova Schar Cancer Center, Fairfax, VA USA; 2 Department of Surgery, MD Anderson Cancer Center, Houston, TX USA; 3 Department of Surgery, University of Wisconsin Hospital and Clinics, Madison, WI USA

**Keywords:** Gastric cancer, Gastroesophageal junction cancer, Gastric adenocarcinoma, Immunotherapy, Pathologic complete response

## Abstract

**Background:**

Perioperative therapies for gastric and gastroesophageal junction (GEJ) adenocarcinoma have improved significantly in recent years, especially for mismatch-repair deficient (dMMR/MSI-H) tumors. Pathologic complete response (pCR) rates are now established which has raised the question as to whether surgery can be omitted.

**Methods:**

This article summarizes the debate between two gastric cancer experts supporting each side of the argument on the possibility of omitting surgery.

**Results:**

Total, proximal, and esophagogastrectomy remain highly morbid procedures that still carry significant risk of mortality, particularly in older patients and at low-volume centers. Patients with dMMR/MSI-H gastric and GEJ adenocarcinoma have a pCR rate to neoadjuvant immunotherapy of 60% and those with mismatch repair intact (MMR-intact) tumors experience pCR to neoadjuvant chemo-immunotherapy with a rate of approximately 20%. The ability to assess for clinical complete response in gastric and GEJ adenocarcinoma has limitations, however, and there is a lack of long-term data to support omission of surgery.

**Conclusion:**

pCR rates are established and durable in gastric and GEJ adenocarcinoma. Omission of surgery is a consideration in patients with dMMR/MSI-H tumors with cCR to immunotherapy. Surgery remains the standard-of-care for patients with MMR-intact tumors but patients should be counseled about the possibility of pCR in shared decision making.

## Background

With advances in chemotherapy and immunotherapy, non-operative management (NOM) and organ preservation are increasingly being incorporated into treatment algorithms for a variety of malignancies, such as rectal adenocarcinoma and esophageal squamous cell carcinoma.^1,2^ Although surgical resection with lymphadenectomy currently plays a critical step in curative-intent treatment for non-metastatic gastric cancer, there is a significant morbidity associated with gastric resection, particularly total gastrectomy.

In terms of perioperative therapies relevant to microsatellite stable (MSS) gastric adenocarcinoma, we have had significant progress over the past 20 years. In 2006, the MAGIC trial regimen of cisplatin, epirubicin, and 5-fluorouracil was established as the standard-of-care perioperative therapy regimen, which yielded a pathologic complete response rate of approximately 5% and a 5-year overall survival of 36%.^3^ In 2019, the FLOT4 study was published, which compared the MAGIC regimen to 5-fluorouracil, oxaliplatin, and docetaxel (FLOT) perioperatively. FLOT yielded a pCR rate of 16% and 5-year overall survival of 45%, establishing it as the new standard-of-care at that time.^4^ Finally in 2025, the Matterhorn trial, which evaluated the addition of durvalumab to FLOT, demonstrated a 19% pCR rate and 2-year overall survival of 70%.^5^ This study defined our current standard-of-care perioperative therapy regimen. For dMMR/MSI-H gastric adenocarcinoma, immune-checkpoint inhibitors have consistently shown pathologic complete response (pCR) in the majority of patients treated.^6,7^ Although no phase III trials have compared surgical resection to NOM yet, organ preservation is increasingly being explored and offered for select patients.

Below, we present a debate on the role of omitting surgery for gastric and gastroesophageal junction (GEJ) cancers based on current evidence. Dr. Kaitlyn Kelly, Professor of Surgery at University of Wisconsin Hospital & Clinics will argue in favor of  NOM for gastric and GEJ adenocarcinoma, while Dr. Brian Badgwell, Professor of Surgery at the University of Texas MD Anderson Cancer Center, will argue against NOM.

## PRO

### Introduction

The treatment of gastric and GEJ adenocarcinoma has changed significantly over the past 20 years. Most of those changes were related to perioperative therapies as described above, and to a lesser extent to surgery. Even though minimally invasive techniques are now routinely employed in gastrectomy and esophagogastrectomy, and proximal gastrectomy with double-tract reconstruction (PG-DTR) can be performed as an alternative to total gastrectomy for proximal cancers, postoperative morbidity, and even mortality, remain quite high. This is particularly of concern for elderly patients, who experience postoperative mortality rates of approximately 5% at high-volume centers and up to 17% at low-volume centers.^5,8^ While these metrics have improved with minimally invasive techniques and attempts at centralization to high-volume centers, long-term quality of life effects that are a direct result of the anatomic changes made in surgery, such as chronic dumping, nutritional deficiencies, malnutrition, along with associated sequelae, such as osteopenia and osteoporosis, and anxiety and depression remain unchanged, raising the question of whether there is a role for NOM in gastric and GEJ adenocarcinoma.^9^

Regarding microsatellite instability (MSI-high) which is a feature in approximately 20% of Western gastric cancer cases, we have consistently seen that neoadjuvant immunotherapy (IO) yields a pCR rate of approximately 60%.^6,7^ This is the subset in which NOM is currently under prospective investigation. These introductory data are summarized in Table 1.

**Table 1:** Subsets of gastric/GEJ adenocarcinoma and likelihood of pCR

Trial	Year	Regimen (control vs. experimental)	Disease type	Design	pCR rate	Survival
NEONIPIGA	2024	Nivolumab + ipilimumab (neo)	MSI-H gastric/GEJ	Phase II / single arm	59%	Pending
INFINITY	2024	Tremelimumab + Durvalumab (neo)	MSI-H gastric/GEJ	Phase II / single arm	60%	Pending
MAGIC	2006	Surgery alone v ECF (peri)	Gastric/GEJ	Phase III RCT	≈5%	5-year OS: 23% vs. 36%
FLOT4	2019	FLOT v ECF/ECX	Gastric/GEJ	Phase III RCT	6% v 16%	5-year OS: 36% vs. 45%
DANTE	2024	FLOT v FLOT + atezolizumab (peri)	Gastric/GEJ	Phase II/III RCT	15% v 24%	Pending
MATTERHORN	2025	FLOT v FLOT + Durvalumab (peri)	Gastric/GEJ	Phase III RCT	7% v 19%	2-year EFS: 59% vs. 67% 2-year OS: 76% vs. 70%

#### Current Supporting Evidence

**Fig. 1:**
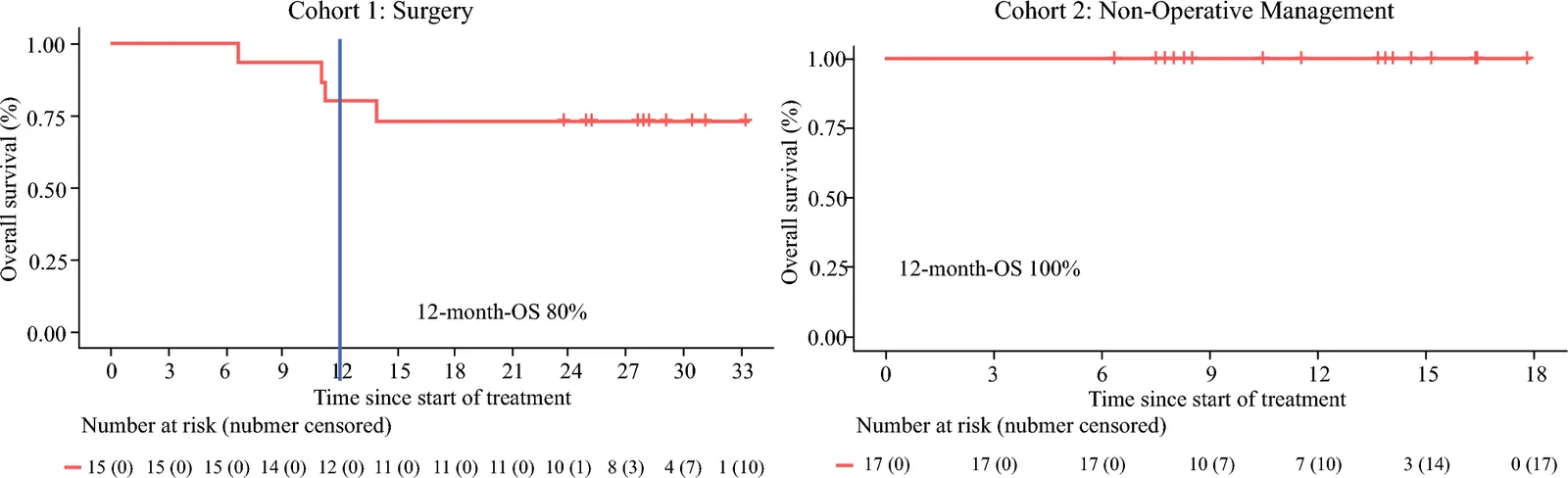
Kaplan-Meier survival curves from the Infinity trial demonstrating 14% perioperative mortality in cohort 1, and 100% 1-year survival in cohort 2, in which surgery was omitted

There is currently very limited data on NOM in GEJ and gastric adenocarcinoma, but the Infinity trial is one that provides some insight regarding MSI-H/dMMR patients. This was a prospective trial for clinical T2 or greater, any N MSI-H/dMMR patients treated with upfront tremelimumab and durvalumab.^7^ The 18 patients in cohort 1 underwent surgery regardless of clinical response, whereas in cohort 2, also consisting of 18 patients, clinical complete responders were observed. In cohort 1, one patient withdrew consent, one had progressive disease, two with cCR refused surgery, and 14 underwent surgery per protocol, nine of whom had pCR (60%). In cohort 2, five patients had surgery owing to toxicity or incomplete clinical response, and 13 had cCR and were observed. Survival curves for these two cohorts are shown in Fig. 1. The operative mortality in cohort 1 was 14% while no patients in cohort 2 experienced death in the follow-up period, with 100% 1-year survival. Similarly in the GERCOR-NEONIPIGA trial of 32 MSI-high patients who received neoadjuvant ipilimumab and nivolumab followed by surgery, the only death or recurrence event in the trial was a postoperative death from a cardiac event on postoperative Day 3.^6^ While this data is limited, it highlights that surgery can harm clinical complete responders with MSI-H/dMMR GEJ and gastric cancer.

In the MSS population, pCR rates are not as high but are real and are established to be approximately 20%, especially in patients with PD-L1 expression.^5^ In this group, we have essentially no data on NOM, making this commentary easy to write. We do, however, have clinical judgement and shared decision making. We do know that a pCR to chemoimmunotherapy is durably associated with improved survival. This was not the case for chemoradiation, which can successfully eradicate the primary tumor, but does not treat micrometastatic disease.^10^ It is important to point out that the survival improvement seen with FLOT + Durvalumab over FLOT in the Matterhorn trial had nothing to do with surgery. It was solely due to the addition of IO to the FLOT regimen and a subsequent analysis showed that a pCR to FLOT + Durvalumab was more robustly associated with event free survival than a pCR to FLOT (hazard ratio 0.29 (0.08–0.96).^5^ We also know that thorough assessment for cCR with EGD/EUS, bite-on-bite biopsies, fine-needle aspiration of lymph nodes, cross-sectional imaging, and potentially minimal residual disease testing, the rate of missed residual disease is acceptable and expected to be approximately 10–15%.^2^

**Fig. 2:**
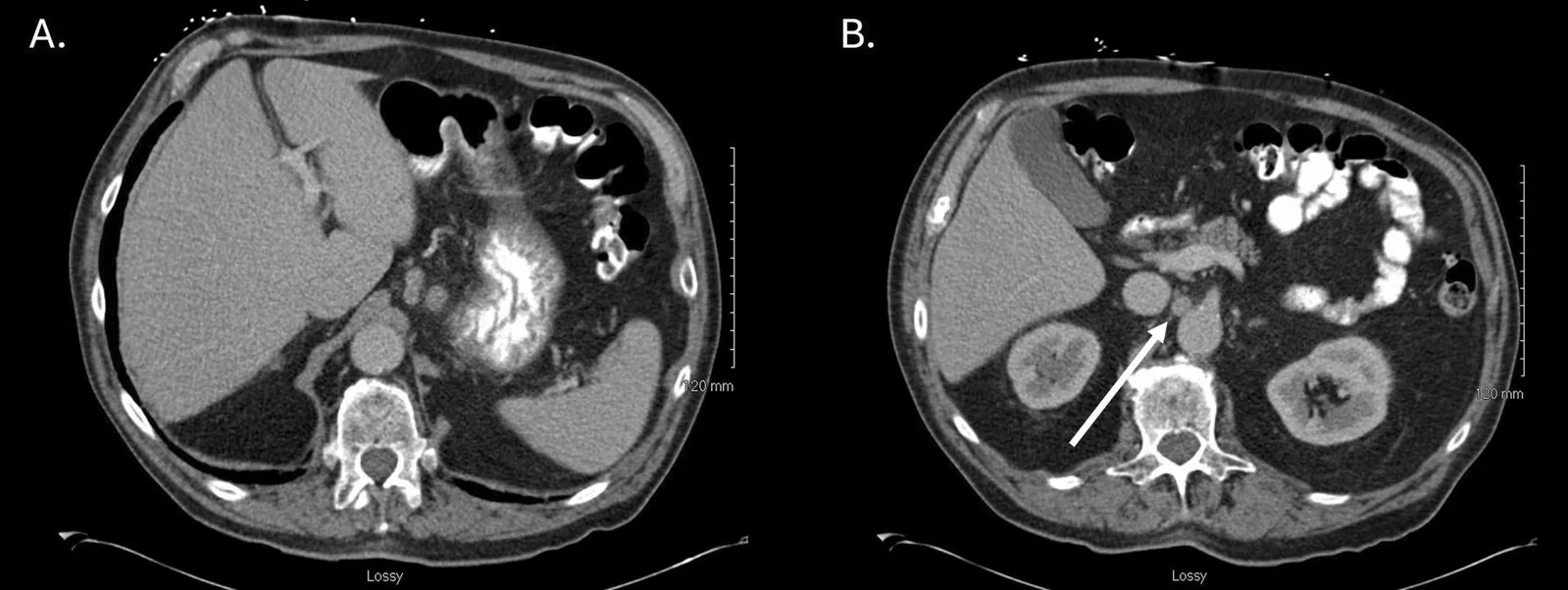
Baseline CT images depicting locally-advanced gastric cancer in an octogenarian with peri-gastric adenopathy (**A**) as well as an enlarged lymph node near the superior mesenteric artery (**B**, arrow)

Given the paucity of data on NOM, I will proceed to brief case reports to demonstrate how I utilize this current information in the care of real patients. Case #1 was an 80-year-old man who presented in 2023 with MSS proximal gastric cancer. At that time, he had a bulky tumor and regional nodal disease on computed tomography (CT) (Fig. 2A). He had a combined positive score (CPS) for PD-L1 of 5% and was initiated on systemic therapy with FOLFOX and Nivolumab at a referring center. He had a cCR and was told he needed a gastrectomy. On review of his baseline, pre-treatment scans, this patient had had an enlarged lymph node near the superior mesenteric artery (Fig. 2B), outside of the modified-D2 field, and he had never undergone a staging laparoscopy or peritoneal washings. Most importantly, this patient’s goal was to maintain high quality of life and he wanted to avoid gastrectomy if at all possible. Considering his degree of response and that he was very likely under-staged at presentation, we made the plan for continuing single agent IO and observation. This patient is now >4 years out from diagnosis, has no evidence of disease (NED), and is off of all therapy.

**Fig. 3:**
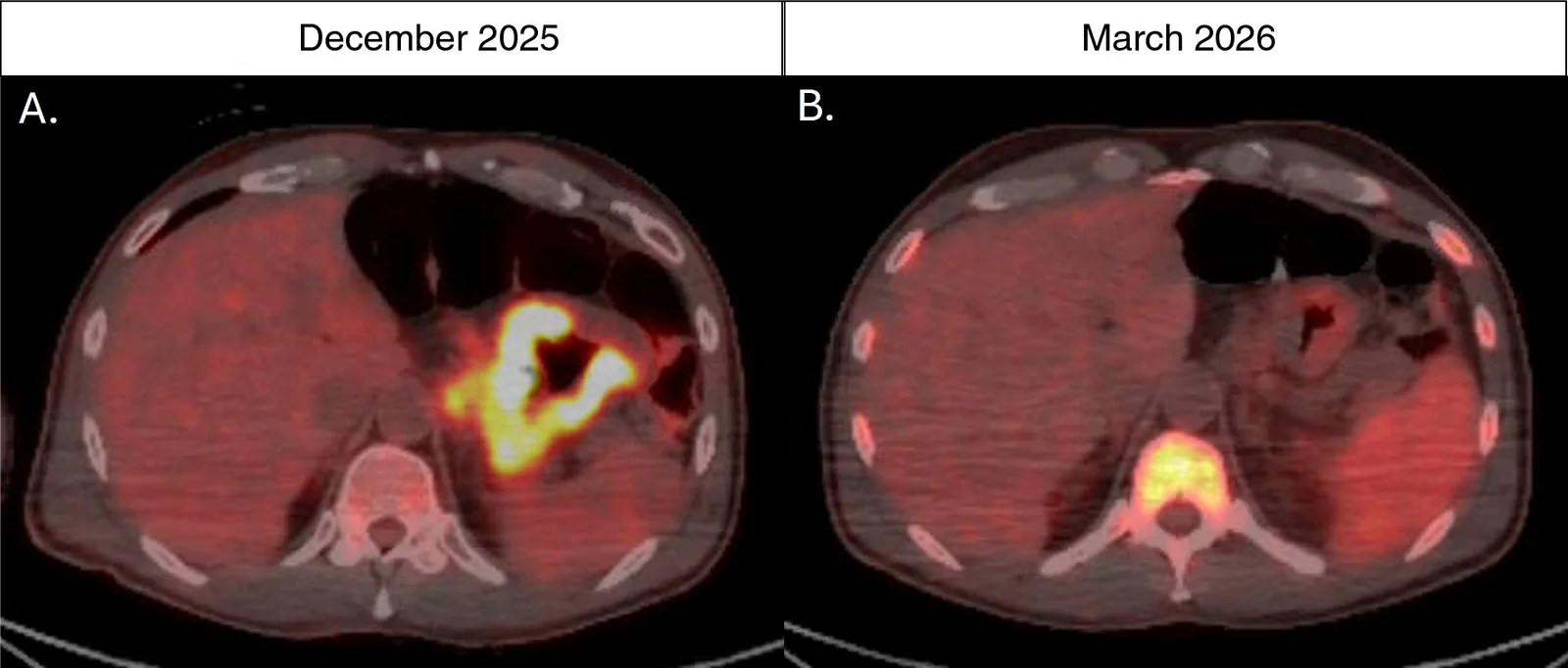
Pre-treatment (**A**) PET/CT scan from a 51-year-old man who presented with poorly-differentiated gastric adenocarcinoma with linitis plastica. He received FLOT + durvalumab and had a marked clinical response but remained with gastric wall thickening and surround adenopathy on post-treatment PET (**B**)

**Fig. 4:**
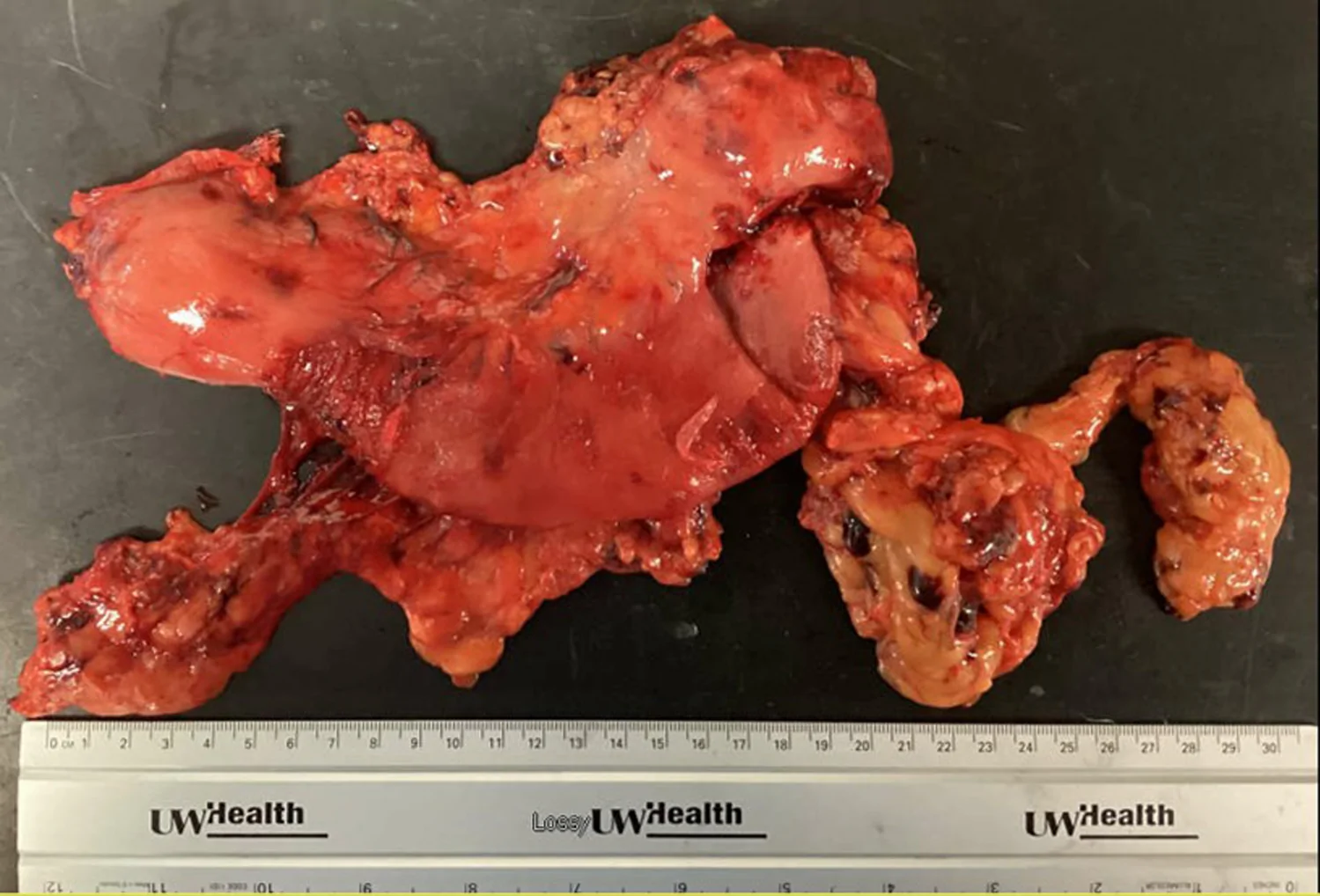
Gross photograph of the stomach from Fig. 4 which appeared markedly abnormal but showed pCR to FLOT + durvalumab. Pathology revealed treatment related changes, including mucosal erosion, fibrosis, and hemorrhage. A total of 89 lymph nodes were examined, of which 27 had treatment effect

Case #2 is a 51-year-old patient who presented in 2025 at a referring center with linitis plastica. Biopsy confirmed diffuse type gastric adenocarcinoma with signet ring cells and a PD-L1 CPS of 5%. In addition to diffuse wall thickening, the patient had bulky perigastric adenopathy (Fig. 3A). His workup included CT and PET/CT but no staging laparoscopy and he was initiated on FLOT + durvalumab. He tolerated therapy well and had a dramatic radiographic response, although not a cCR as his stomach still appeared abnormally thickened (Fig. 3B). He was referred for surgery at that time. Due to concern for under-staging at baseline, we performed a staging laparoscopy with washings which revealed no evidence of active or previously treated gross peritoneal disease, and washings were negative. His stomach still looked and felt abnormal with serosal injection and thickening. Given his young age, excellent performance status, and findings at laparoscopy, we recommended him to undergo total gastrectomy. The patient expressed a wish to avoid surgery but after thorough discussion about the likelihood of residual disease being 80%, he agreed to proceed. His family members, one of whom was a healthcare provider, also very much wished for him to proceed. He underwent a robotic total gastrectomy in early 2026 and his pathology revealed a pCR despite the stomach still appearing markedly abnormal grossly (Fig. 4). The stomach was described as having “treatment related changes including mucosal erosion, fibrosis, and hemorrhage.” A total of 89 lymph nodes were examined, of which 27 had treatment effect. This patient is overall doing well and did not have complications, but struggles with the fact that he had a pCR when it was his overall preference to avoid surgery.

Case #3 was a 67-year-old man with Lynch Syndrome who presented in 2025 with MSI-H/dMMR gastric cancer. The patient had undergone previous total abdominal colectomy for colon cancer and Whipple procedure for pancreatic cancer and had a BMI of 21 at presentation. The gastric mass was near his gastro-jejunal anastomosis. He wished to avoid another operation and received ipilimumab and nivolumab with cCR. Given his multiple prior cancers and prior abdominal surgeries, surgery was omitted. His follow-up is limited at one year, but he remains clinically NED.

These cases highlight the considerations to be made in considering NOM of GEJ and gastric adenocarcinoma. NOM is already accepted for elderly patients with MSI-H/dMMR tumors who would need total, proximal, or esophagogastrectomy because surgery poses a very real risk of harm in these patients, the majority of whom will have pCR to IO. For MSS patients, the decision to omit surgery is more difficult due to lack of data and lower likelihood of pCR. However, for patients who present with locally advanced (especially ≥cT3 or node positive) disease and who did not have complete baseline staging with laparoscopy and washings, and go on to have cCR to chemo-immunotherapy, it is very questionable that halting a therapy that is working to put them through major surgery, with high risks and significant quality of life changes, is the right thing to do. Table 2 summarizes factors to consider to assist in clinical decision making in patients with ≥cT3 or node positive GEJ or gastric adenocarcinoma who have had cCR to therapy.

**Table 2:** Factors to consider to aid in clinical decision making about surgery for patients with locally advanced GEJ or gastric adenocarcinoma and cCR to therapy

	Favor surgery	Consider-NOM
Patient factors		
Age and performance status	Fit for surgery	Elderly / high risk
Disease factors		
Expected pCR rate based on histology and therapy received	MSS / PD-L1- and/or FLOT: **7–16%**	MSI-high with IO: **60%** MSS PD-L1+ with FLOT+ Durva: **20%**
Surgery known to be curative-intent?*	Yes	No
Operation required	Distal subtotal gastrectomy	Total / proximal / esophagogastrectomy

Bold test in the table highlights established pCR rates

*Baseline stage confirmed by laparoscopy with washings

## Future

In this era of treatment of GEJ and gastric adenocarcinoma, it is more important than ever that patients be appropriately staged at baseline. Chemotherapy is no longer the same for those with localized disease versus those with metastatic disease in whom PD-L1, HER-2, and Claudin 18.2 are potential targets and in whom surgery should be omitted because it will not be curative. Compliance with staging laparoscopy as a stand-alone procedure is poor and needs to be more widely adopted moving forward so that patients receive appropriate and optimal therapy.

Future studies also need to focus on the optimal duration of a neoadjuvant regimen in patients who demonstrate response and wish to avoid surgery. Surgery to remove the stomach and/or GEJ is a big ask for any patient just to learn that their disease was already eradicated. This is especially true for elderly patients or those with significant comorbidities. Now that FLOT + durvalumab is standard-of-care, we will be increasingly faced with the scenario of cCR and will need to take patient goals into account in decision making about gastrectomy. Additionally, as demonstrated in case #2 above, patients can have a pCR without a cCR. This highlights that we as surgeons need to ask ourselves and our patients how we will feel if we proceed with an operation and find a pCR. While it is good news and predicts the best prognosis for someone who started with locally advanced gastric cancer, there is no reason to think that gastrectomy in the setting of pCR does anything for the patient other than provide prognostic information, and render them stomach-less. If an individual patient with marked clinical response wishes to avoid surgery and take the chance that they have had a pCR, that option should be presented to them. Finally, we need to follow patients who elect to omit surgery prospectively to understand long-term outcomes if surgery is omitted, as in case #1 above.

## CON

### Introduction

The good news is that chemoradiotherapy allows for selective surgery in patients with gastroesophageal squamous cell cancer. The bad news is that this approach only applies to a minority of our patients. Current NCCN guidelines for esophageal cancer also include a pathway to observation for adenocarcinoma treated with preoperative chemoradiotherapy, although esophagectomy is preferred.^11^ MSI-H/dMMR tumors are one bright spot due to the consistent findings of high response rates to IO and even pCR rates on the order of 60%. Outside of MSI-H/dMMR tumors, however, we are nowhere near the ultimate goal of systemic therapy that is so effective the primary tumor and all circulating tumor cells are eradicated permanently. For our older, frail patients with comorbid conditions prohibitive for total gastrectomy or esophagectomy, we do not even have a good non-surgical option to reliably get us 3–5 years of tumor control.

#### Current Supporting Evidence

We should first address the most exciting marker for avoiding surgery for resectable gastroesophageal tumors—MSI testing. The enthusiasm for patients with MSI-H/dMMR tumors was evident in NCCN guidelines for gastric cancer in 2024 whereby patients with localized disease treated with immune checkpoint inhibitors and clinical complete response (based on imaging and upper endoscopy) were recommended to undergo observation only.^12^ The surveillance frequency is not specifically defined for these tumors, but standard guideline follow-up is every 6 months for 2 years and then annually for up to 5 years. Because we have only had routine testing for MSI status since approximately 2019, we should acknowledge we do not have reliable long-term data. Other areas of concern include a less than 100% pathologic complete response, unknown optimal regimen choice between single versus doublet immune checkpoint inhibition, optimal length of treatment, and reliability of surveillance. For an older patient requiring a total gastrectomy, the decision-making is easy to avoid surgery. But for a young patient who would only require a subtotal gastrectomy is the risk and long-term surveillance worth avoiding a low-risk surgery? Our expert colleagues at the NCCN must have recognized these concerns as current 2026 guidelines now recommend observation or surgery.^13^

There have also been more modest advancements in response rates for systemic chemotherapy for microsatellite-stable tumors. The FLOT regimen has shown a path CR rate of 7% with triplet chemotherapy compared with only 2% with doublet therapy. Now with the incorporation of immunotherapy for resectable gastric cancer, the pathologic complete response rate is 19% for Durvalamab-FLOT and 13% for Cis/5FU with pembrolizumab.^5,14^ These pathologic complete response rates are good but not high enough to consider avoiding surgery, and the durability of a complete clinical response for microsatellite-stable tumors is unknown. With a 2-year overall survival rate of 76% with Durvalamab-FLOT, we may be getting close to a 70% cure rate after resection. Personally, I am hesitant to lose this high chance for cure unless we have a very high clinical complete response rate and reliable testing to ensure pathologic complete response.

The improvements in survival from targeted therapy have been more elusive. In the randomized phase II HERFLOT trial comparing FLOT to FLOT with trastuzumab and pertuzumab for Her2+ tumors, the pathologic complete response rate was an impressive 35%.^15^ The overall survival difference at 24 months favored the experimental arm at 84% versus 77%, but noted higher rates of diarrhea and leukopenia and did not advance to phase III. Another Phase II trial failed to advance to phase III when comparing FLOT to FLOT with ramicurimab.^16^ The pathologic CR rate was 18% with ramicurimab but included more grade 3 and higher adverse events with no significant difference in survival.

## Future

The trials above demonstrate the danger in utilizing pCR rates as a surrogate for survival. The trials of organ preservation for esophageal cancer also have uncovered limitations in active surveillance and how this might adversely impact long-term outcomes for those that even undergo resection without metastatic disease. With trial concepts in development based on a predicted pCR rate of 30% for organ preservation, I have strong concerns over the omission of surgery. The length of preoperative therapy is also important due to our limitations in identifying a response to therapy. The promise of circulating tumor DNA (ctDNA) may come to fruition in contributing to active surveillance and response to therapy. Let’s not forget medical oncology is not the only discipline obtaining advancements in outcomes, as surgery is becoming safer due to minimally-invasive approaches, enhanced recovery, and overall better perioperative care. The equation to evaluate avoiding surgery is unknown, but will at least include the variables of cCR rates, pCR rates, efficacy of active surveillance, surgical morbidity, and quality of life. For microsattelite-stable tumors in a patient with acceptable surgical risk the current risk-benefit evaluation clearly favors including surgery. We should focus our initial efforts of NOM on the patients at high risk for surgical intervention, which we frequently encounter in our aging population. Based on the rapid improvements in bispecifics, antibody drug conjugates, targeted therapy, and certainly immunotherapy the future is bright but we need surgeon involvement to ensure that we do not miss the chance for cure.

## Summary

As we have outlined above, NOM of gastric and GEJ adenocarcinoma is not ready to become standard-of-care for all-comers. Critical questions remain before organ preservation can be widely adopted in gastric cancer. Standardization of surveillance protocols, including incorporation of ctDNA, should be developed to ensure appropriate monitoring for all patients forgoing surgical resection. Appropriate duration and regimen of ICI-therapy is still debated, and head-to-head trials are needed to determine optimal treatment for dMMR/MSI-H patients. Further validation of the correlation between clinical and pathologic complete response is also needed, particularly in MSS patients.

Despite these questions, data from the INFINITY trial would support careful consideration of omission of surgery in a select subset of patients, specifically older, frailer patients with MSI-H/dMMR tumors for whom resection would necessitate a total gastrectomy, and who demonstrate a cCR to neoadjuvant immunotherapy.^7^ However, NOM should be pursued only at high-volume gastrectomy centers with a rigorous surveillance protocol and a combined team of medical oncologists, surgical oncologists, and gastroenterologists following the patients on surveillance. Patients intended for NOM should be designated at the time of diagnosis to ensure appropriate staging workup is completed and that patients can adequately comply with the surveillance protocol. It should also be recognized that NOM may not always be successful. In a limited cohort of patients undergoing NOM in the INFINITY trial, one patient experienced recurrence, but was able to successfully undergo salvage surgery.^7^ This highlights the importance of surgeons remaining engaged and involved with the patients trialing organ preservation

Arguments against NOM highlight the limited specificity of cCR for pCR, as a majority of patients deemed to have achieved a cCR were found to harbor residual viable disease either in the primary tumor site or in lymph nodes.^17^ Additionally, long-term follow-up for non-operative cohorts is limited, ranging from 11.5–28 months, so little is known about the durability of NOM. Finally, surgical outcomes continue to improve with the utilization of minimally-invasive techniques, and gastrectomy offers an excellent long-term prognosis with 5-year OS of approximately 61% for MSS patients and 88% for MSI-H/dMMR patients.^18^

## Conclusions

The decision to forgo surgical resection in gastric adenocarcinoma should be made as part of a multidisciplinary discussion. The NCCN guidelines recommend considering observation in MSI-H/dMMR patients with a cCR, while surgery remains standard-of-care for all other patients. Further research and clinical trials are needed to definitively determine the role of NOM in non-metastatic gastroesophageal cancer.
